# Mechanisms of Resistance to Targeted Therapy in Epidermal Growth Factor Receptor (EGFR)- and Anaplastic Lymphoma Kinase (ALK)-Mutated Non-small Cell Lung Cancer: Molecular Basis, Emerging Biomarkers, and Therapeutic Strategies

**DOI:** 10.7759/cureus.115685

**Published:** 2026-09-02

**Authors:** Sneha Dhillon, Heena Rathod, Sagar Dhillon

**Affiliations:** 1 Radiation Oncology, All India Institute of Medical Sciences, Rajkot, IND; 2 Radiation Oncology, M. P. Shah Medical College, Jamnagar, IND; 3 Ophthalmology, JIET Medical College and Hospital, Jodhpur, IND

**Keywords:** alk, drug resistance, egfr, liquid biopsy, non-small cell lung cancer, precision oncology, tyrosine kinase inhibitors

## Abstract

Currently, with the spectacular achievement of the tyrosine kinase inhibitors (TKIs), non-small cell lung cancer (NSCLC) carrying mutations of the gene epidermal growth factor receptor (EGFR) and rearrangements of the gene anaplastic lymphoma kinase (ALK) is becoming a paradigm of precision oncology. While there has been great progress in the field of progression-free survival (PFS) and longer-term clinical results have been achieved, resistance sets in, and the long-term impact of these therapies is limited. A summary of the molecular mechanisms of acquired resistance to EGFR- and ALK-targeted drugs is provided here, ranging from secondary changes in the target kinases, via activation of other signaling pathways, to epithelial-mesenchymal transition, intratumoral tumor heterogeneity, and interactions with the tumor microenvironment. For early detection of resistance, emerging biomarkers such as liquid biopsies, circulating tumor DNA, exosomes, and biomarkers, multi-omics characterization and AI-assisted prediction models are discussed. The review also covers an overview of the new treatment strategies on the horizon, including sequential TKI therapy, combination targeted therapy, fourth-generation inhibitors, antibody-drug conjugates, bispecific antibodies, and personalized precision medicine. Finally, current clinical trials, future directions, and challenge/limitations are discussed, focusing on the need for adaptive molecular monitoring and a customized approach to overcome resistance and maximize long-term outcomes in EGFR- and ALK-mutated NSCLC.

## Introduction and background

In spite of significant advances in the diagnosis and treatment of cancer, non-small cell lung cancer (NSCLC) is the most common cause of cancer-related death all around the globe, and approximately 85% of all lung cancer cases are NSCLC. It has significant molecular and clinical heterogeneity and different therapeutic responses and patient outcomes. Platinum peptides are the standard of care in advanced NSCLC; however, that management has been changed significantly by new understanding that disease-specific oncogenic mutated driver genes have been identified, which began precision oncology. The discovery of molecularly targeted therapies that specifically target abnormal signaling pathways that have been implicated in the initiation and progression of tumors has brought about significant advances in clinical outcomes and transformed the paradigm of various types of therapy, from histology-based to genotype-driven [1].

Global burden of NSCLC

Lung cancer is still a major burden in the world and has millions of new cases every year and millions of deaths caused by cancer. NSCLC represents almost 85% of all lung cancers and consists of various histological types of cells such as adenocarcinoma, squamous cell, and large-cell. While still strongly associated with tobacco smoking, the number of never smokers is now reported, and environmental pollution, occupational carcinogens, genetic susceptibility, and lifestyle risk factors are expected to be contributing factors to the count of new cases among never smokers. With the advent of low-dose computed tomography (LDC) for the diagnosis of disease, and multidisciplinary therapy, many advances have been made, but there remains a significant number of patients who present with poor long-term survival rates due to either locally advanced or metastatic disease [2].

Precision oncology has changed the face of clinical management of NSCLC. Promising breakthroughs in next-generation sequencing and a more thorough molecular characterization of tumors now make it easier to identify actionable genomic abnormalities prior to the start of treatment. It's like a molecular classification; it allows us to use the molecular entity and not necessarily just the pathological features to tailor the therapy. As such, targeted therapies have recognized superior objective response rates, progression-free survival, utilization of quality of life, as well as lower systemic toxicity versus traditional cytotoxics, making molecular testing an integral part of today's management of NSCLC [3].

Driver mutations in NSCLC

In NSCLC, several genomic alterations have been found, some of the most important therapeutic targets being activation of epidermal growth factor receptor (EGFR) and rearrangements of the receptor gene of anaplastic lymphoma kinase (ALK). These EGFR mutations, in particular the L858R mutations (in exon 21) and the deletions (in exon 19), result in activated constitutive receptor tyrosine kinase signaling and subsequent metastasis formation, apoptosis resistance, proliferation, and angiogenesis. Similarly, oncogenic fusion proteins created by chromosomal rearrangements, such as EML4-ALK fusion proteins (primarily the EML4-ALK oncogenic fusion protein), are able to cause perpetual activation of ALK without the presence of a ligand and cause the formation of the tumor [2,4].

These driver mutations have been proven to be of great significance in thoracic oncology and have implications for clinical practice. New guidelines for the use of molecular testing have been recommended, suggesting routine molecular testing for patients who are planning to initiate first-line systemic therapy in advanced NSCLC for EGFR mutations, ALK rearrangements, and other actionable biomarkers. These molecular stratifications exist to help select the most active targeted drugs and to improve the likelihood of therapeutic benefit and minimize over-exposure to those drugs less likely to help [3, 4].

Development of targeted therapy

Over the last 20 years, the arrival of tyrosine kinase inhibitors (TKIs) compared to the situation before 2002, before the approval of gefitinib, has changed the scenario of EGFR-mutated and ALK-mutated NSCLC. However, the first-generation reversible EGFR inhibitors, like gefitinib and erlotinib, had unprecedented clinical efficacy compared to chemotherapy and were limited in their scope of application by the problem of acquired resistance. Other inhibitors with irreversible and dual activity, such as the second-generation afatinib and dacomitinib, broadened erythroblastic oncogene B (ErbB) receptor family inhibition, and third-generation inhibitors are directed specifically against the T790M resistance mutation and optimized for central nervous system penetration, particularly osimertinib. In a similar fashion, ALK-directed treatment progressed quickly over a decade, from the first-generation crizotinib to more potent second-generation ALK inhibitors, alectinib, ceritinib, and brigatinib, of which the latter shows excellent intracranial activity and is effective against multiple resistance variants, and the third generation, lorlatinib. The continuous evolution in the field of precision medicine is reflected in more recent preclinical and early clinical development of fourth-generation inhibitors that are able to overcome the development of compound resistance mutations (Figure 1) [3-5].

**Figure 1:**
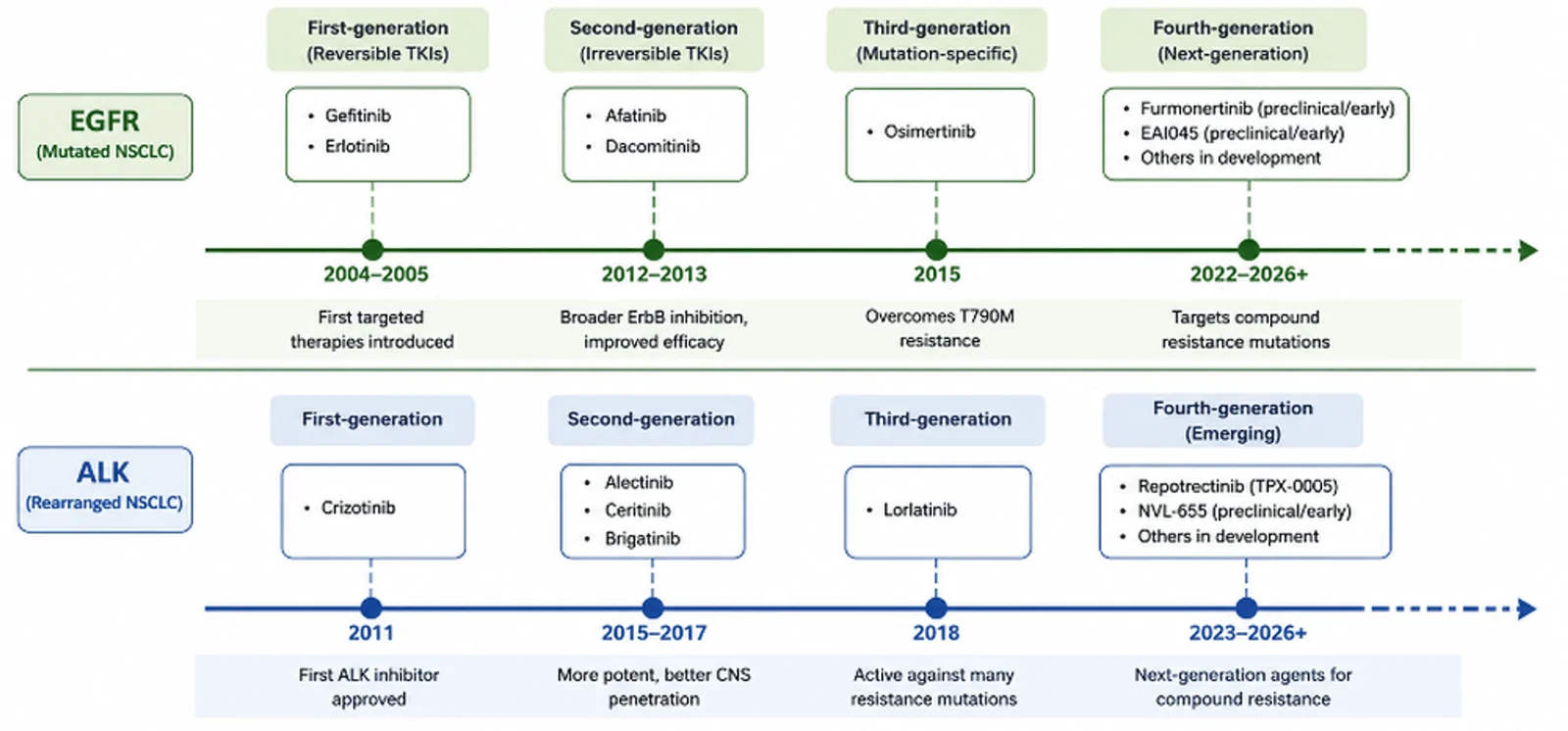
Evolution of targeted therapies for EGFR- and ALK-mutated NSCLC The figure was created by the authors using PowerPoint (Microsoft, Redmond, Washington). EGFR - epidermal growth factor receptor; ALK - anaplastic lymphoma kinase; TKI - tyrosine kinase inhibitor; ErbB - erythroblastic oncogene B; CNS - central nervous system

Clinical challenge of acquired resistance

Although targeted drug therapy has been a tremendous success in the clinic, most patients will experience therapeutic resistance. Resistance occurs in various ways, such as by secondary mutations in the kinase domain, turning on alternative complementary signaling pathways, epithelial-mesenchymal transition, histological change, intratumoral heterogeneity, and changes in the interaction with differences in the tumor microenvironment. Among the most critical technical limitations of precision oncology these days are the ability of these adaptive mechanisms to (re-)activate oncogenic signals without challenging drug action, the generation of resistance to the drug in terms of patient-specific acetate resistance, and the persistence of therapeutic response [4,5].

Scope and objectives of the review

The purpose of this review was to go in depth about the underlying molecular mechanisms of resistance to targeted therapies in EGFR- and ALK-mutated NSCLC. It aims to highlight the state-of-the-art information about the intracellular signaling changes, genomic events found in the resistant cell, and the interactions that occur between the tumor microenvironment and the resistant cell, predictive biomarkers, and new therapeutic strategies to overcome resistance mechanisms. Further, the review discusses directions of future precision oncology efforts, which may serve as a basis for operative individualized treatment approaches aiming to improve the long-term clinical outcome of patients at the onset of the treatment of molecularly defined NSCLC.

## Review

Molecular biology of EGFR and ALK signaling pathways

Constitutive activation of receptor tyrosine kinase (RTK) signaling pathways that control key cell functions such as proliferation, survival, differentiation, metabolism, angiogenesis, and metastasis is responsible for the pathogenesis of EGFR- and ALK- mutated NSCLC. These signaling networks are important as the underpinning of targeted therapies and the molecular infrastructure to understand therapeutic sensitivity and the development of drug resistance. Although both EGFR- and ALK- mutations activate several common downstream pathways, there are significant differences between them with regard to the molecular events and clinical and biological consequences (Figure 2) [6,7].

**Figure 2:**
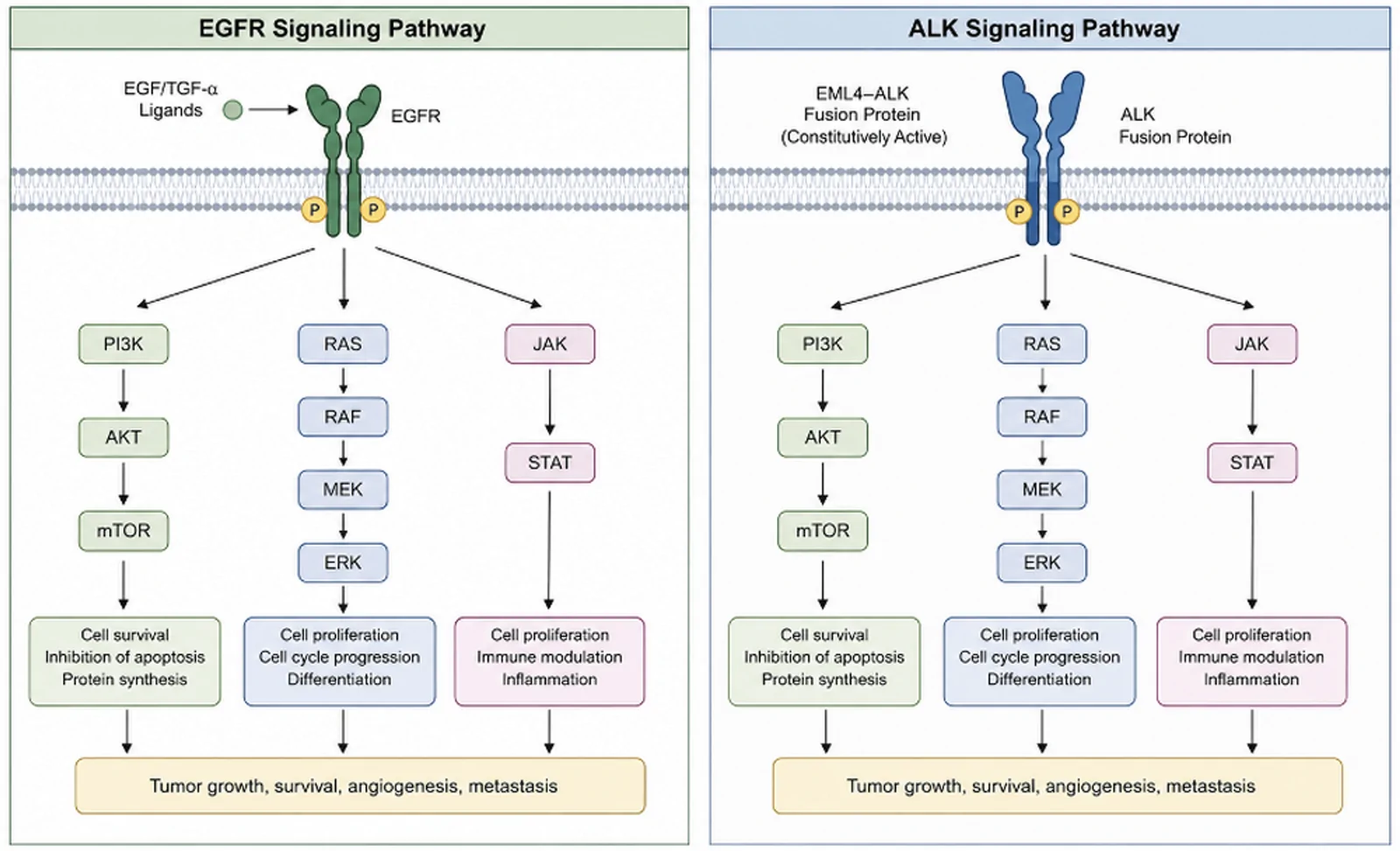
EGFR and ALK signaling pathways with downstream molecular cascades The figure was created by the authors using PowerPoint (Microsoft, Redmond, Washington). EGFR - epidermal growth factor receptor; PI3K - phosphoinositide 3-kinase; AKT - protein kinase B; mTOR - mechanistic target of rapamycin (historically called mammalian target of rapamycin); RAS - rat sarcoma; RAF - rapidly accelerated fibrosarcoma; MEK - mitogen-activated protein kinase kinase; ERK - extracellular regulated protein kinase; JAK - Janus kinase; STAT - signal transducer and activator of transcription; ALK - anaplastic lymphoma kinase

EGFR Signaling Pathway

Epidermal growth factor receptors (EGFR) are a member of the ErbB family of receptor tyrosine kinases that are found on the plasma membrane of cells. The receptor homo/heterodimerization resulting from ligand binding leads to autophosphorylation of tyrosines located on the intracellular domains of the receptor and recruitment of several adaptor proteins, initiating, under normal physiological conditions, multiple pathways of intracellular signaling mechanisms. Activated EGFR triggers activation of three main pathways, the phosphoinositide 3-kinase and protein kinase B (PI3K/AKT) pathway, which helps cells survive and inhibits their death; the mitogen-activated protein kinase (MAPK) cascade, which controls cell proliferation, differentiation and progression through the cell cycle; and the Janus kinase/signal transducer and activator of transcription (JAK/STAT) pathway, which is involved in the regulation of the immune system, inflammation, proliferation and progression of tumors. Mutations of the EGFR that occur in the normal receptor allow for their continuous activation and signaling for malignant transformation, without requiring the binding of a lymphocyte or action of the ligand [6-8].

ALK Signaling Pathway

The ALK rearrangements are due to inversion events at the ALK gene, which result in the production of fusion proteins, which are, in most cases, constitutively active fusion proteins of EML4-ALK. Several distinct variants of EML4-ALK fusion have been described that all show different patterns of biology, kinase activation, sensitivity to therapy, and resistance to therapy. Activated ALK fusion proteins are able to activate several downstream signaling pathways, such as PI3K/AKT, MAPK, and JAK/STAT, that can lead to uncontrolled cellular proliferation, inhibition of apoptosis, angiogenesis, increased metastatic potential of cells, and tumor maintenance. However, these signaling pathways are constantly activated, and ALK rearrangements can continue to fuel some patients with NSCLC [8,9].

Molecular Differences Between EGFR- and ALK-Driven Tumors

While dependence on the signaling pathway of receptor tyrosine kinases (RTKs) is common to both EGFR-mutated and ALK-mutated tumors, these tumors are drastically different in terms of underlying molecular biology and clinical features. While tumors positive for ALK are due to base-chromosomal rearrangements that create constitutively active fusion proteins, EGFR-mutated tumors have been found to have activating point mutations or short in-frame deletions at the kinase domain of the EGFR gene. These fundamental differences at this molecular level may impact downstream signaling, the process of metastasis, drug resistance, and sensitivity to subsequent generations of targeted therapy. Also, there are other differences between ALK-positivity and EGFR mutations; EGFR mutations are more prevalent in Asian countries, in females and in younger patients compared to ALK-positive mutations, which are more prevalent in never-smokers and patients with adenocarcinoma histology [9, 10].

Importance of Oncogene Addiction

EGFR-mutated and ALK-mutated NSCLC are a paradigm of 'oncogene addiction': these tumor cells require continual activation of a dominant oncogenic signaling pathway for their survival and proliferation. Inhibition of these driver kinases by pharmacological means, therefore, leads to rapid responses by the tumor cells and remarkable clinical successes. Meanwhile, this 'grazing' generates strong selective pressure for resistant cell clones that recover a signal for activation of a downstream pathway, either by second-site mutations, or by signaling avoidance, or by phenotypic adaptation. Hence, knowledge related to how these different biological events govern the process of 'oncogene addiction' is still crucial for developing inhibitors for the next generation and rational combination inhibitors and precision treatment strategies that will delay or overcome acquired resistance to inhibitors [10,11].

Current targeted therapies in EGFR- and ALK-mutated NSCLC

Through the discovery of targeted tyrosine kinase inhibitors (TKIs) that target EGFR mutations and ALK rearrangements, the management of NSCLC has been revolutionized by the tumor's localization. These inhibitors are known to selectively target oncogenic kinases necessary for the growth of the tumor cell and offer the promise of higher rates of treatment response, longer progression-free survival (PFS), improved quality of life, and significantly reduced systemic toxicity over current conventional chemotherapy agents. Several generations of TKIs have been developed on the basis of the simple science of the drugs they target with the aim of combating resistance mutations to the drug and improving control over the disease in the central nervous system (CNS) (Table 1) [12,13].

**Table 1:** Comparison of currently approved EGFR and ALK inhibitors The table was compiled by the authors [12-17]. EGFR - epidermal growth factor receptor; Ex19del - exon 19 deletion; ErbB - erythroblastic oncogene B; ALK - anaplastic lymphoma kinase; MET - MNNG HOS transforming gene/ mesenchymal-epithelial transition factor; ILD - interstitial lung disease

Drug	Generation	Target mutation	FDA approval	Median PFS	Major resistance mechanisms	Common adverse effects
Gefitinib	First	EGFR Ex19del, L858R	Yes	9–11 months	T790M, MET amplification	Rash, diarrhea
Erlotinib	First	EGFR Ex19del, L858R	Yes	10–13 months	T790M, bypass activation	Rash, diarrhea, hepatotoxicity
Afatinib	Second	EGFR and ErbB family	Yes	11–13 months	T790M, HER2 amplification	Diarrhea, stomatitis, rash
Dacomitinib	Second	EGFR and ErbB family	Yes	14–15 months	T790M, MET amplification	Rash, diarrhea, paronychia
Osimertinib	Third	EGFR Ex19del, L858R, T790M	Yes	18–19 months	C797S, MET amplification	Diarrhea, QT prolongation, ILD
Crizotinib	First	ALK rearrangement	Yes	10–11 months	ALK mutations, MET activation	Visual disturbances, edema
Ceritinib	Second	ALK rearrangement	Yes	16–18 months	G1202R, bypass signaling	Gastrointestinal toxicity
Alectinib	Second	ALK rearrangement	Yes	34–35 months	ALK compound mutations	Constipation, myalgia
Brigatinib	Second	ALK rearrangement	Yes	24–29 months	Compound ALK mutations	Hypertension, CPK elevation
Lorlatinib	Third	Resistant ALK mutations	Yes	27–36 months	Compound ALK mutations	Hyperlipidemia, cognitive effects

EGFR Tyrosine Kinase Inhibitors

The most successful ones to date have been EGFR tyrosine kinase inhibitors (TKIs); there are a number of ATP-competitive reversible inhibitors that are effective against the EGFR activating mutations, predominantly L858R in exon 21 and exon 19 deletions. These agents have shown to be better at achieving PFS outcomes than platinum-based chemotherapy, but eventually acquired resistance occurs, most frequently by a gatekeeper mutation on T790M [12,13].

A multitude of ErbB receptor family members are targeted by second-generation EGFR inhibitors such as afatinib and dacomitinib, which are irreversible inhibitors. In addition, they are more likely to be wild-type inhibitors and reduce the emergence of resistance in a certain portion of patients; however, their more intense inhibition of the wild-type EGFR can cause more dermatological and gastrointestinal toxicity [13,14].

The third-generation EGFR inhibitor osimertinib selectively inhibits both the activating mutations and the T790M resistance mutations in the EGFR and spares wild-type EGFR. Evidence to date has preferred the use of osimertinib as the first-line treatment for EGFR-mutated advanced NSCLC because of its higher efficacy, high CNS penetration, and good toxicity profile [14,15]. Now, new-generation fourth-generation EGFR inhibitors, including allosteric inhibitors like BLU-945 and BBT-176, are being tried clinically against the more complex resistance mutations, like the EGFR C797S and multiple EGFR mutants [15].

ALK Tyrosine Kinase Inhibitors

The treatment of ALK-mutated NSCLC has undergone dramatic changes over the last 10 years. The first-generation ALK inhibitor drug crizotinib was notably clinically effective; however, it has impaired CNS penetration and has lost its efficacy due to the occurrence of secondary ALK mutations and bypass signaling [15,16].

The development of second-generation inhibitors such as alectinib, brigatinib, and ceritinib was developed in an effort to circumvent crizotinib resistance. It is these agents that have higher potency in kinase activity, higher efficacy in the CNS, and longer progression-free survival (PFS), which makes alectinib and brigatinib an excellent choice of initial treatment in many guidelines [16]. Lorlatinib is a third-generation inhibitor with a wide spectrum of activity, including against multiple ALK resistance mutations, including the highly refractory G1202R mutation, and has excellent blood-brain barrier penetrance for the brain metastatic patient [16,17].

Two new fourth-generation ALK inhibitors, TPX-0131 and NVL-655, are in clinical development that target compound ALK mutations that occur after the use of TKIs in sequence. These novel agents may be the next step in precision oncology in achieving prolonged disease control following resistance to existing low-of-label agents [17].

Resistance to EGFR-targeted therapy

Despite the often dramatic benefit of prognosis enhancement that EGFR tyrosine kinase inhibitors result in, resistance to the drug does develop in almost every person who is treated with an EGFR TKI for a long time to treat EGFR-mutated NSCLC. Different mechanisms are available to counteract this resistance and are extremely diverse; some are intrinsic changes in the composition of the molecule(s) before treatment, some are acquired, or changes of the genome or epigenetics of the molecule(s) that occurred during treatment with therapeutic agents (Figure 3). A significant development in understanding these resistance pathways and the rational development of next-generation inhibitors and combination therapy with liquid biopsy technology has come in recent years [18,19].

**Figure 3:**
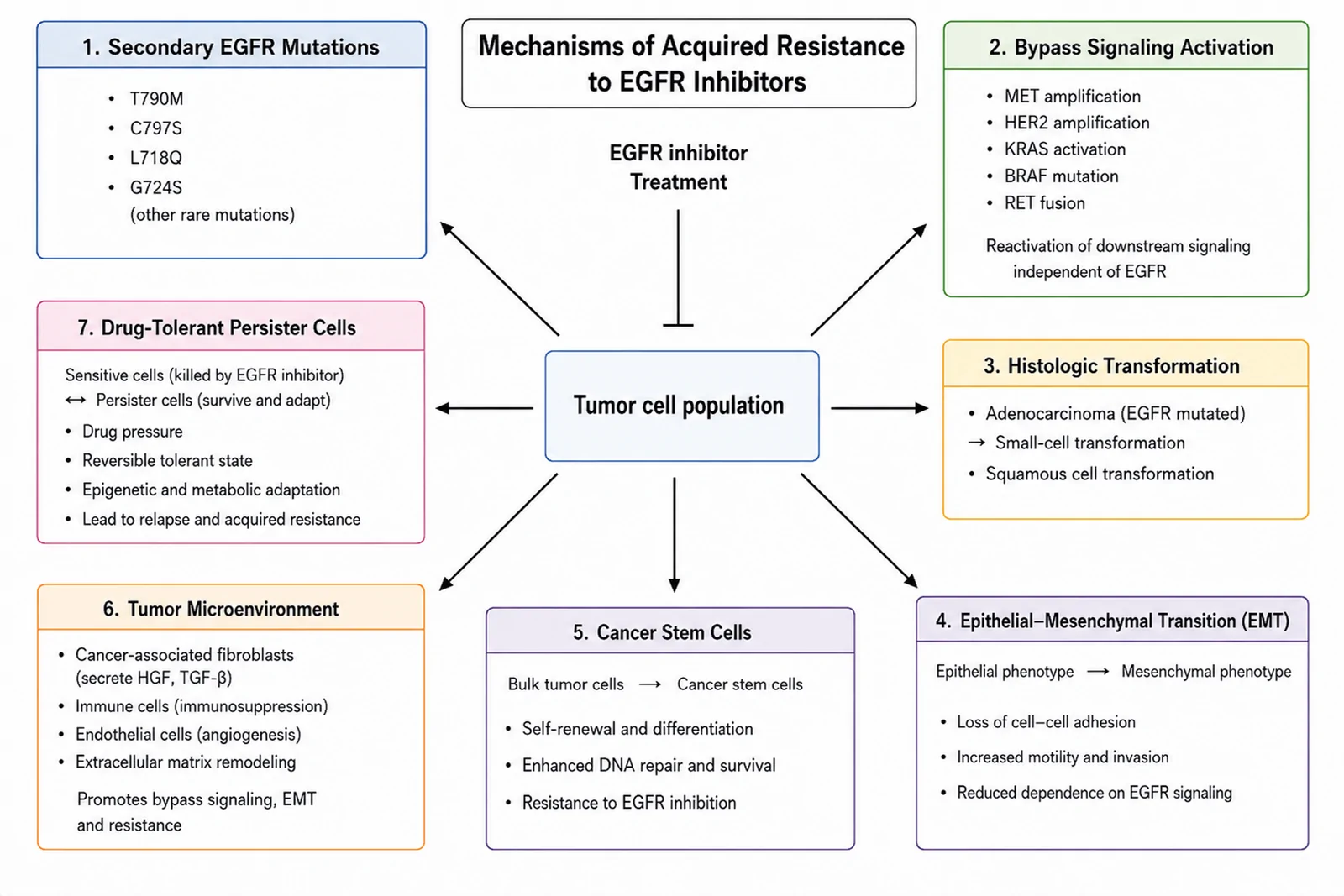
Mechanisms of acquired resistance to EGFR inhibitors The figure was created by the authors using PowerPoint (Microsoft, Redmond, Washington). EGFR - epidermal growth factor receptor; HER2 - human epidermal growth factor receptor 2; MET - MNNG HOS transforming gene/ mesenchymal-epithelial transition factor; KRAS - kirsten rat sarcoma viral oncogene homolog; BRAF - B-Raf proto-oncogene, serine/threonine kinase

Primary Resistance

Primary (intrinsic) resistance is when there is an EGFR mutation but no detectable clinical effect. EGFR mutations that are rare, such as exon 20 insertion and rare mutations of the EGFR kinase domain, have lower sensitivity to conventional EGFR TKIs. Furthermore, other somatic alterations in TP53, PIK3CA, RB1, MET, or other survival pathways can diminish therapeutic efficacy by providing cancer cells with alternative survival pathways that are independent of EGFR signaling [18,19].

Secondary EGFR Mutations

The most well-characterized mechanism of resistance is acquired secondary mutations inside the domains of the EGFR kinase. In cases of resistance after first- and second-generation TKIs, resistance is explained, in about half of the cases, by the acquisition of the T790M dimer system gating mutation that alters the affinity for ATP and decreases the ability of the TKI to bind. Other mutations, however, which have appeared in the process of treatment with osimertinib, led to interference with the irreversible interaction with osimertinib and restored kinase activities, such as C797S, L718Q, and G724S mutations [19,20].

Bypass Signaling Activation

The most trivial mechanism of the avoidance of EGFR receptor inhibition, which is frequently seen, is that the signaling downstream of the EGFR becomes stimulated by other signaling pathways that the tumor cells are capable of using; these other signaling pathways can sustain the downstream signaling and cell proliferation even though effective blockade of EGFR has occurred. Most frequently seen bypasses are MET amplification, HER2 amplification, KRAS activation, BRAF mutations, and RET gene fusions. Such molecular modifications may result in the reactivation of PI3K/AKT and MAPK pathways that can bypass the need for EGFR pathway and allow progression of the tumors despite the use of EGFR inhibitors [20,21].

Histologic Transformation

Another clinically important resistance mechanism is phenotypic transformation. However, in very few cases, EGFR-mutated adenocarcinomas could turn into small-cell lung cancer (SCLC) or, rarely, a squamous cell carcinoma, without the mutated EGFR being lost. The altered morphology of these tumors revealed new biological characteristics, with most having other therapeutic requirements than additional EGFR inhibition [21].

Epithelial-Mesenchymal Transition (EMT)

The hallmark of EMT is a loss of epithelial markers and an up-regulation of the expression of mesenchymal proteins, spontaneous cell mobility, and invasiveness. EMT renders cells less reliant on the EGF pathway and can add to resistance by engaging other survival pathways and enhancing metastatic potential [21,22].

Cancer Stem Cells

Cells that initiate the cancer, called cancer stem cells, are a small number of cells that have the power to self-renew; targeted therapies are ineffective. These cells evade EGFR inhibition by activating developmental signaling pathways, by metabolic reprogramming, and by greater DNA repair to ultimately lead to disease recurrence after initial response to therapy [22].

Tumor Microenvironment (TME)

TME is an important therapeutic resistance factor; the interactions between cancer-associated fibroblasts, immune cells, endothelial cells, and the extracellular matrix are complex in nature. The cytokines in the medium, such as hepatocyte growth factor and transforming growth factor-β, stimulate bypass signaling, immune suppression, and EMT, resulting in resistance to EGFR inhibitors [22,23].

Drug-Tolerant Persister Cells

Apart from the EGFR-inhibitor-resistant subpopulation with a permanently altered genome, there is a subpopulation that can survive for a long time without embarking on irreversible resistance, namely a drug-tolerant persister subpopulation. Epigenetic reprogramming, metabolic adaptation, and activation of stress-response in these cells cause temporary survival under therapeutic stress. Over time, persistent genomic alterations might occur in persister cells that lead to true, acquired, and irreversible resistance, and clinical relapse [23].

Mechanisms of resistance to ALK-targeted therapy

Although patients with ALK-mutated NSCLC have a much-improved prognosis since the advent of ALK- TKIs, resistance will emerge in patients treated with these drugs. As with targeting of EGFRs, ALK inhibitors have a large number of resistance mechanisms, both genetic and epigenetic, and microenvironmental, which allow for re-revitalization of oncogenic signaling in the presence of permanently ongoing inhibition of ALK. Even though second- and third-generation ALK inhibitors delayed disease progression, using successive inhibitors putrains selective pressure that leads to the generation of more and more complex resistance changes such as compound kinase mutations, activation of bypass signaling pathways, epithelial plasticity, and tumor heterogeneity (Figure 4). Adopting a proper knowledge of these mechanisms is crucial to create new-generation inhibitors and individualized therapeutics (Table 2) [24,25].

**Figure 4:**
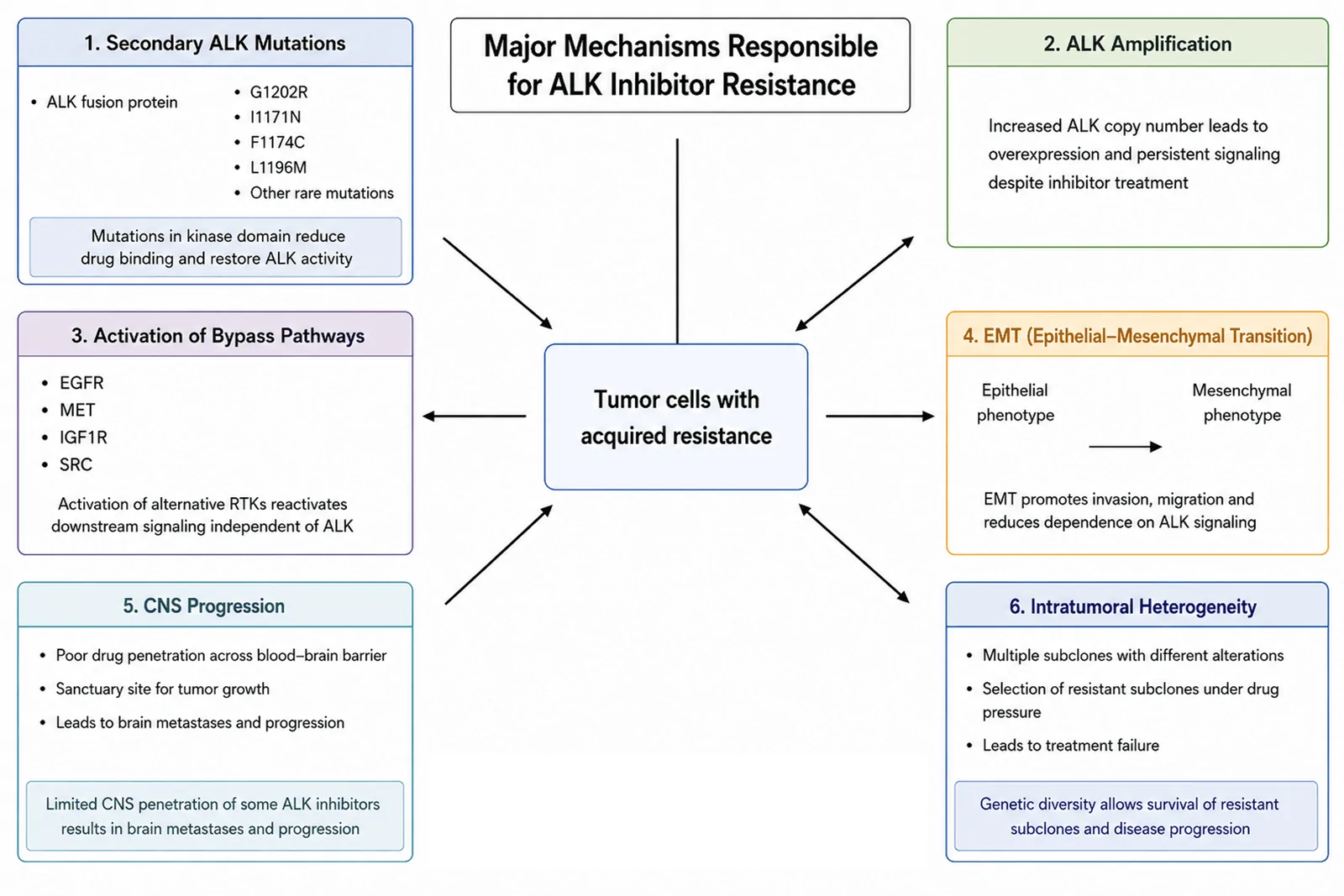
Major mechanisms responsible for ALK inhibitor resistance The figure was created by the authors using PowerPoint (Microsoft, Redmond, Washington). ALK - anaplastic lymphoma kinase; RTK - receptor tyrosine kinases; EMT - epithelial-mesenchymal transition; CNS - central nervous system

**Table 2:** Major mechanisms of resistance to ALK-targeted therapy The table was compiled by the authors [24,25]. ALK - anaplastic lymphoma kinase; EMT - epithelial-mesenchymal transition; CNS - central nervous system; EGFR - epidermal growth factor receptor; MET - MNNG HOS transforming gene/ mesenchymal-epithelial transition factor; IGF1R - insulin-like growth factor 1 receptor; PI3K - phosphoinositide 3-kinase; AKT - protein kinase B; MAPK - mitogen-activated protein kinase

Resistance mechanism	Representative alterations	Biological consequence	Potential therapeutic strategy
Secondary ALK mutations	G1202R, I1171N, F1174C, L1196M	Reduced ALK inhibitor binding	Next-generation ALK inhibitors
ALK amplification	Increased ALK copy number	Persistent ALK signaling	Potent ALK inhibition or combination therapy
Bypass pathway activation	EGFR, MET, IGF1R, SRC	Reactivation of PI3K/AKT and MAPK pathways	Dual-targeted combination therapy
EMT	Loss of E-cadherin, increased vimentin	Increased invasion and drug resistance	EMT-targeted and combination therapies
CNS progression	Brain metastases	Pharmacologic sanctuary site	CNS-penetrant ALK inhibitors
Intratumoral heterogeneity	Multiple resistant subclones	Mixed therapeutic response	Precision medicine guided by serial molecular profiling

Secondary ALK Mutations

Most resistance to ALK inhibitors is due to secondary mutations of the ALK kinase domain. These mutations cause an alteration in either the ATP binding pocket or the conformation of the kinase, thus decreasing the drug's binding affinity. A variety of mutations are associated with resistance, but it is presumed that G1202R is most important, as it renders resistance to a variety of second-generation ALK inhibitors. Other frequent mutations that have been found include I1171N with reduced sensitivity to alectinib, F1174C with increased kinase activity that is associated with ceritinib resistance, and L1196M (gatekeeper mutation), which was the original cause of crizotinib resistance. Most resistance mutations are also dependent on the type of ALK inhibitor used, a strong indication that repeat molecular profiling is important in the course of the disease [24,25].

ALK Amplification

Another key resistance mechanism is that of amplification of the ALK fusion gene. The more copies of ALK, the more the ALK fusion protein is expressed, and the more uninterrupted the ALK pathway's "signaling" despite the anti-ALK drug. Although not seen as frequently, when the kinase mutation occurs, ALK amplification can be present as well, and may in part explain resistance to the various generations of ALK inhibitors. This adaptive ability of the tumor cells can be further emphasized under chronic targeted therapy through genomic amplification [25,26].

Activation of Bypass Pathways

One of the most common mechanisms by which tumor cells that challenge ALK inhibitors still grow is by activation of alternative receptor tyrosine kinase pathways responsible for downstream signaling for proliferation independent of ALK. Signaling via EGFR, MET, IGF1R, and SRC has long been known to be associated with therapeutic resistance. The pathways culminate with common intracellular effectors that are regulated by both and restore cell survival, proliferation, and metastatic potential while ALK is still inhibited, including PI3K/AKT or MAPK (mitogen-activated protein kinase). As a result, a number of clinical trials are currently investigating dual treatment, which involves inhibition of ALK and bypass pathways [26,27].

Epithelial-Mesenchymal Transition (EMT)

In addition to genetic changes, an important non-genetic mechanism to resist drugs is phenotypic transformation, which can occur by the process of epithelial-mesenchymal transition (EMT). In EMT, cells of the epithelial tumors take on characteristics of a mesenchymal cell, such as enhanced ability to resist apoptosis, invade tissues, and migrate. These alterations not only decrease the reliance on ALK signaling, but they also stimulate a number of alternative pathways, which decrease the effect of targeted therapy [27].

Central Nervous System (CNS) Progression

In ALK-mutated NSCLC, the progression of the disease is most often in the central nervous system. The low CNS permeability of first-generation ALK inhibitors has helped the development of CNS metastases while systemic disease has been controlled. Newer drugs like alectinib, brigatinib, and lorlatinib have very good activity in the CNS, but development of this disease compartment is a significant hurdle for both clinicians and pharmacokinetics; this is due to the availability of resistant cellular clones, the adaptation of the cells in the CNS microenvironment, and limitations in pharmacokinetics in the CNS [28].

Intratumoral Heterogeneity

Intratumoral heterogeneity may be significant as it raises the potential of the presence of multiple genetically distinct subclones and thus poses a major challenge to targeted therapy in individual patients. Selective pressure of ALK inhibition gives rise to resistant subpopulations with distinctive molecular changes; subsequent emergence and final domination of resistant subpopulation(s) contribute to the progression of the disease. It is a dynamic evolving process that may also result in the acquisition of compound resistance mutations or heterogeneity of the susceptibility to different treatments in each metastatic lesion; for this reason the need for sequential molecular monitoring for individual treatment, both with tissue biopsy and circulating tumor DNA (ctDNA) analysis. [28,29].

Tumor microenvironment and immune modulation in targeted therapy resistance

It has been known that resistance against targeted therapy not only is generated by genetic modifications in the tumor, but also that the so-called tumor microenvironment (TME) plays a substantial role in it. The TME contains cancer-associated fibroblasts, immune cells, endothelial cells, extracellular matrix factors, as well as other factors such as cytokines and hypoxic areas, which control tumor development, immune escape, angiogenesis, and therapy responsiveness. Adaptive resistance means that dynamic interactions between malignant cells and the stromal microenvironment elicit activation of more signaling pathways/round the killing agent and rescue the targeted cells (Figure 5). Thus, modulation of TME has become a valuable approach to bypass resistance to EGFR- and ALK-directed therapies [30,31].

**Figure 5:**
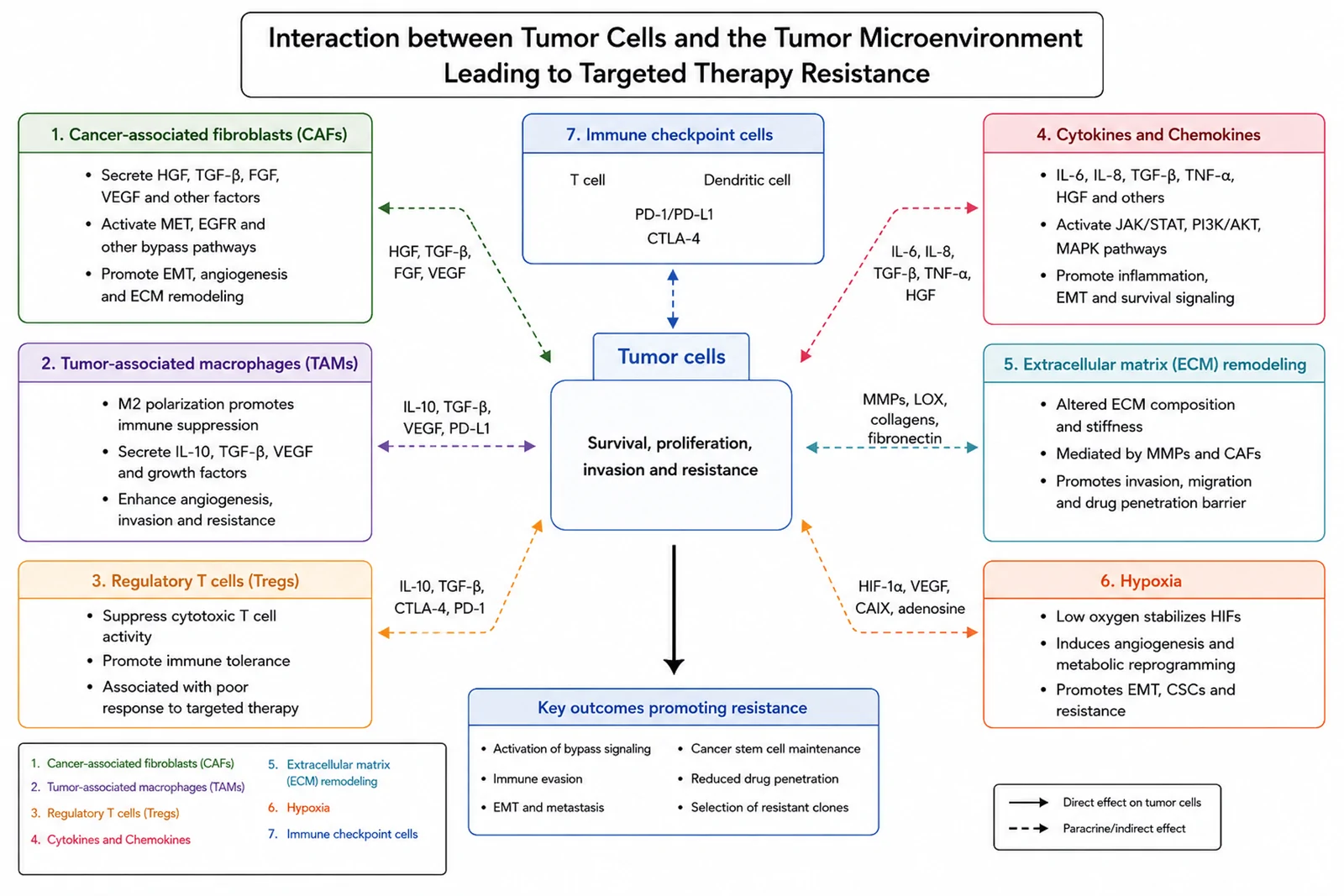
Interaction between tumor cells and the tumor microenvironment The figure was created by the authors using PowerPoint (Microsoft, Redmond, Washington). EMT - epithelial-mesenchymal transition; ECM - extracellular matrix; JAK/STAT - Janus kinase/signal transducer and activator of transcription; MMP - matrix metalloproteinases; CAF - cancer-associated fibroblast; HIF - hypoxia-inducible factor; CSC - cancer stem cell

Cancer-Associated Fibroblasts

The stromal microenvironment is closely associated with therapeutic resistance, and the cancer population is infiltrated by stromal cells including cancer-associated fibroblasts (CAFs). To activate bypass signaling pathways that do not involve EGFR or ALK, CAFs release many growth factors: hepatocyte growth factor (HGF), transforming growth factor-β (TGF-β), fibroblast growth factors, and vascular endothelial growth factor (VEGF). These paracrine signals help to recruit EMT, angiogenesis, remodeling of the matrix, and ongoing tumor proliferation despite the presence of continued kinase inhibition [30,31].

Tumor-Associated Macrophages

These comprise the so-called tumor-associated macrophages (TAMs), which are significant in aspects of host immune responses and also in targeted therapy resistance. M2-polarized macrophages are immunosuppressive and secrete cytokines like interleukin-10, TFG-β and induce the angiogenic mediation using VEGF. Furthermore, TAMs promote tumor invasion, metastatic spread, and activate survival signal pathways, which contributes to the less long-term effectiveness of targeted therapeutic agents [31,32].

Regulatory T Cells

Regulatory T cells (Tregs) in the tumor microenvironment (TME) promote tumor cell tolerance and suppress anti-tumor immune function, particularly the function of the anti-tumor effector cells called cytotoxic T lymphocytes. The high infiltration is associated with a low response rate to treatment, favorable tumor progression, and inferior OS. Also, they are immunosuppressive and enable to develop resistance to tumor cells during targeted therapy [32].

Cytokines

A big chunk of adaptive resistance is understood as a mediator known as the cytokine network. Multiple downstream signaling pathways, including JAK/STAT, PI3K/AKT, and MAPK pathways, are activated by these cytokines (IL-6, IL-8, TGF-β, TNF-α, and HGF) and induce cell survival, inflammation, EMT, and therapeutic resistance. Chronic cytokine signaling also leads to increased communication between cancer cells and other stromal cells, helping the cancer cells respond to therapeutic stresses and continued survival [32, 33].

Extracellular Matrix Remodeling

During tumor progression, the extracellular matrix (ECM) can be remodeled by the action of the matrix metalloproteinases (MMPs) and the stromal fibroblasts. As ECM composition and stiffness change, so too do the capacities of cell adhesion, mechanotransduction, migration, and the availability of drugs (growth factors), and the increased degree of invasive behavior of a cell decreases the penetration of drugs into tumor tissue. An important molecular mechanism of resistance consists of these alterations in the structure, which contribute to resistance to targeted therapies [33].

Hypoxia-Mediated Resistance

Hypoxia is another important factor of the resistant microenvironment. As a result of deprivation of oxygen, hypoxia-inducible factors (HIFs) are activated, which causes a boost of angiogenesis, metabolic reprogramming, immune suppression, and activation of alternative survival pathways. Hypoxia also promotes epithelial-mesenchymal transition and cancer stem cell maintenance, leading to continued disease in the presence of good oncogenic driver inhibition. Hypoxia-induced signaling has hence emerged as a new opportunity to gain information about how to better tackle targeted therapy efficacy [33,34].

All these add weight to the notion that therapeutic resistance may be driven by a complex reciprocal interaction between malignant cells and the microenvironment. Combined inhibition of oncogenic drivers and the microenvironment has become a focus in the future of treatment strategies, which aims to approach more clinical adaptation and delay disease progression [34].

Emerging biomarkers for early detection of resistance

Early identification and characterization of resistance mechanisms are essential for guiding subsequent treatment decisions and optimizing personalized therapeutic strategies in EGFR- and ALK- mutated NSCLC. Resistance mechanism identification is therefore crucial and an up-front approach in precision oncology in EGFR- and ALK-mutated NSCLC (ALK-Lung). A traditional tissue biopsy is the most reliable method for molecular profiling; however, due to the problem of tumor heterogeneity and inaccessibility, repeated sampling may be non-invasive, costly, and time-consuming. Therefore, the use of minimally invasive biomarkers that allow dynamic monitoring of tumor evolution has become of great interest. In the last years, new technologies in liquid biopsy, circulating biomarkers, multi-omics approaches, and artificial intelligence (AI) have made it possible to assess molecular changes from therapeutic resistance in real time in order to intervene at an early stage and make a personalized treatment change (Table 3) [35,36].

**Table 3:** Emerging biomarkers predicting resistance in EGFR- and ALK-mutated NSCLC Table is compiled by authors [35 to 44].

Biomarker	Sample source	Clinical application	Advantages	Limitations
Circulating tumor DNA (ctDNA)	Plasma	Detection of resistance mutations, MRD monitoring	High sensitivity, non-invasive	Low abundance in some patients
Cell-free DNA (cfDNA)	Plasma	Tumor burden assessment	Easy serial monitoring	Lower tumor specificity
Circulating tumor cells (CTCs)	Peripheral blood	Disease progression and prognosis	Cellular characterization possible	Low detection rate
Exosomal DNA/RNA	Plasma, serum	Early resistance detection	Stable biomolecules	Isolation standardization required
miRNAs / lncRNAs / circRNAs	Blood, exosomes	Predictive and prognostic biomarkers	High stability	Validation still required
Multi-omics signatures	Tissue and blood	Precision medicine and patient stratification	Comprehensive molecular profiling	High cost and computational complexity
AI-integrated biomarkers	Multi-source datasets	Predictive modeling and treatment selection	Improved predictive accuracy	Requires large validated datasets

Liquid Biopsy

One of the most promising tools for surveillance of resistance to targeted therapy has been liquid biopsy. Circulating tumor DNA (ctDNA) can be used for the detection of resistance-associated mutations, including EGFR T790M and C797S, and small/large molecule ALK resistance mutations long prior to obvious radiologic disease progression. Apart from ctDNA, there is another type of circulating cell-free DNA that can give complementary information regarding the tumor burden, response to treatment, and minimal residual disease (cfDNA). There is only one drawback: repeated tissue biopsies cannot be performed repeatedly to witness longitudinal molecular monitoring, whereas this aspect is compensated by serial liquid biopsy, where intratumoral heterogeneity aspects are taken care of [35-37].

Circulating Tumor Cells

Circulating tumor cells (CTCs) have great potential for tumor metastasis and drug resistance information. Molecular characterization of CTCs offers a unique opportunity to learn about the molecular alterations taking place during disease progression as well as evaluate the epithelial-mesenchymal transition (EMT) and changes in phenotype. Furthermore, quantitative analysis of CTCs has been shown to be associated with therapy response and prognosis, thus providing an exciting means of monitoring the disease by the number of CTCs [37,38].

Exosomal Biomarkers

Exosomes are nanosized extracellular vesicles that are secreted by tumor cells and facilitate the exchange of proteins, lipids, RNA, DNA, and signaling molecules between tumor cells and the tumor microenvironment. The content of exosomes encapsulates molecular features of the primary tumor and, by intercellular communication, dictates drug resistance. Discovery of resistance proteins in exosomes and of mutant DNA parts and regulatory micro-RNAs gives a non-invasive approach to predict failure of therapy as well as disease progression [38, 39].

RNA Biomarkers

Multiple types of RNAs, such as microRNAs (miRNAs), long non-coding RNAs (lncRNAs), circular RNAs (circRNAs) and messenger RNAs, have come forward as potential biomarkers of targeted therapy resistance. These RNA species are expressed in an inappropriate way, and thus they control epithelial-mesenchymal transition, apoptosis, immune evasion, as well as oncogenic signaling pathways regulating resistance. They are appealing targets for minimally invasive molecular diagnostics, due to their presence in blood and other body fluids [39,40].

Multi-Omics Biomarkers

Comprehensive multi-omics profiling is the integration of the genomic, transcriptomic, proteomic, metabolomic, and epigenomic information to gain a comprehensive view of tumor biology. Several molecular layers can be integrated, allowing the determination of complex resistance networks, beyond mere changes in one gene. With multi-omics techniques, it is also possible to achieve better stratification of patients and discovery of new therapeutic targets for a personalized therapeutic approach [40-42].

Artificial Intelligence-Assisted Biomarker Discovery

Large-scale genomic, radiological, pathological, and clinical data are being combined using such artificial intelligence and machine learning algorithms in the realm of biomarker discovery. AI can use machine learning algorithms to identify shared molecular mechanisms associated with therapeutic resistance and create predictive models to forecast response to treatment approaches, which can help inform therapeutic decision-making on a patient-by-patient basis. The combination of AI with multi-omics is expected to lead to further developments of precision oncology due to AI's capacity to be predictive of the targeted therapy choice and outcomes and the ability to enable earlier identification of resistance [42-44].

Resistance management in therapy

Despite acquired resistance, new therapeutic strategies to extend the disease control period have been developed with the aim of prolonging the patient's survival, targeted to patients harboring EGFR- and ALK-mutated NSCLC. Modern strategies for treatment are increasingly based on a combination of molecularly targeted drugs, antibody-based therapeutics, precision medicine-based intervention, and immunotherapy rather than on sequential drug inhibition based on different kinases (Table 4). It is vital to have comprehensive molecular profiling at all stages of the disease since resistance mechanisms require using appropriate therapy [45,46].

**Table 4:** Therapeutic strategies to overcome resistance in EGFR- and ALK-mutated NSCLC The table was compiled by the authors [45-52]. TKI - tyrosine kinase inhibitor; EGFR - epidermal growth factor receptor; MET - mesenchymal-epithelial transition factor; VEGF - vascular endothelial growth factor; ALK - anaplastic lymphoma kinase; HER2/HER3 - human epidermal growth factor receptor 2/3

Therapeutic strategy	Target/Mechanism	Clinical rationale	Current status
Sequential TKI therapy	Emerging resistance mutations	Delays disease progression	Standard clinical practice
EGFR + MET inhibition	MET amplification	Suppresses bypass signaling	Approved/advanced clinical trials
EGFR + VEGF inhibition	Angiogenesis + EGFR	Improves progression-free survival	Clinically validated
Fourth-generation EGFR inhibitors	C797S, compound EGFR mutations	Overcomes osimertinib resistance	Early clinical development
Next-generation ALK inhibitors	Compound ALK mutations	Post-lorlatinib disease control	Clinical trials
Antibody-drug conjugates	HER2/HER3 and other targets	Targeted cytotoxic drug delivery	Expanding clinical use
Bispecific antibodies	Dual receptor inhibition	Prevents bypass pathway activation	Clinical development
Combination immunotherapy	Immune modulation	Enhances anti-tumor immunity	Ongoing clinical trials
Precision oncology and adaptive therapy	Molecular-guided treatment	Individualized resistance management	Emerging standard of care

Sequential TKI Therapy

Managing resistance is still a fundamental principle in the management of TKI-derivatives - to use one TKI after another. Today, the third-generation ALK inhibitor Osimertinib has shown a very high objective response rate (ORR) in patients who have acquired ALK resistance mutations, in whom first- and second-generation ALK inhibitors have failed, and also has activity in ALK wild-type patients who have the T790M mutation. Therefore, a molecular reassessment at progression is vital for other rational sequencing strategies to be possible [45,46].

Combination Targeted Therapy

Combination therapy has become an effective approach towards the bypass pathway activation. Dual EGFR and MET inhibitors have demonstrated clinical activity in MET-amplified tumors that have progressed on EGFR inhibitors. Likewise, dual targeting of both EGFR and VEGF stands out as it inhibits angiogenesis, as well as interferes with the oncogenic signaling pathway, with progression-free survival being increased in some patient subpopulations. Other multi-agent therapies of the HER2, RET and KRAS pathways are being evaluated in clinical trials [46,47].

Fourth-Generation EGFR Inhibitors

There are novel, fourth-generation EGFR inhibitors specifically designed to address these osimertinib-resistant mutations (C797S) and compound EGFR mutations. Many ATP-competitive and allosteric inhibitors are currently in development and are currently undergoing human clinical trials and have been found to be clinically effective with complex patterns of resistance [47,48].

Next-Generation ALK Inhibitors

New ALK inhibitors, such as TPX 0131 and NVL-655, have shown to have strong activity against various compound ALK resistance mutations and have favorable CNS penetration. These agents have been developed to achieve activity against agents resistant to lorlatinib, and represent a step forward in the sequential ALK-targeted treatment concept [48,49].

Antibody-Drug Conjugates

Antibody-drug conjugates (ADCs) are an emerging class of biopharmaceuticals that are targeting the delivery of powerful therapeutic drugs specifically to tumor cells with a lowered level of systemic toxicity using highly specific monoclonal antibodies. Two targeting HER3 (AD031 [49], DS-8214 [50]) as well as two targeting HER2 (AP 1809 (IMMATEK) [50,51]) have demonstrated efficacy in TKI-resistant patients with EGFR-mutated NSCLC.

Bispecific Antibodies

Through their simultaneous action, bispecific antibodies target two independent molecular pathways that are linked to processes of tumor progression. Blocking agents targeting the EGFR and MET (or targeting other complementary receptors) have demonstrated efficacy in blocking bypass signaling and delaying resistance and may prove to be a future therapy for molecular heterogeneity tumors [50].

Combination with Immunotherapy

While immune checkpoint inhibitors (ICIs) have been largely ineffective when used as a single drug in oncogene-driven NSCLC, there is a great deal of effort underway to explore combinations with targeted therapies, anti-angiogenic drugs, or chemistries. These are targeting strategies to overcome immune escape mechanisms associated with acquired resistance towards targeted therapies and enhance anti-tumor immunity [50,51].

Personalized Precision Oncology

The developments in whole-genome sequencing and in serial liquid biopsy, combined with the development of multi-omics analysis, have made true personalization a reality in therapeutic decision-making. Treating cancer using the dynamic molecular state of a tumor as opposed to just the snapshot genomic information is recognized as an important aspect of modern precision oncology treatment selection [51].

Adaptive Treatment Strategies

Adaptive therapeutic strategies are aimed at the dynamic accumulation of molecular data and continual dose/regimen adjustment, depending on the molecular mechanism of resistance. Together with continuing the benefit of the patient in the long run, the use of AI, LIQ, and predictive computational models could potentially enable tailoring of the therapy to a single patient and delay development of resistance, which occurs in patients with both EGFR- and ALK-mutated NSCLC [52].

Future therapeutic landscape

Precision oncology appeared on the scene like a whirlwind and is the product of many exciting pioneering clinical trials that have changed standard practice for people with EGFR- and ALK- mutated NSCLC. These studies give impressive gains in progression-free survival, overall survival, and intracranial control as well as in quality of life, in comparison with conventional chemotherapy. Meanwhile, several research programs are dedicated to testing new inhibitors, rationally combined therapies, and creating strategies applicable to biomarker identification and treatment to avoid acquired drug resistance. When combined, these developments are defining a new future for therapies based on personalized, adaptive, and mechanism-based approaches [53,54].

Landmark Clinical Trials

Targeted therapy approaches for NSCLC have greatly changed due to several key clinical trials. The FLAURA trial found that osimertinib improves both PFS and OS over the use of first-generation EGFR TKIs in the treatment of EGFR-mutated NSCLC. AURA showed how effective osimertinib was in those patients who had the secondary EGFR T790M mutation who had unresponsiveness to previous-generation EGFR inhibitors. For ALK-mutated disease, ALEX demonstrated superiority for alectinib in both systemic and intracranial PFS, and lorlatinib proved to be a highly effective first-line ALK inhibitor with its excellent CNS and systemic efficacy in the CROWN study. Combination targeted therapies that work on resistance mechanisms such as EGFR and MET signaling were recently introduced in the MARIPOSA and PAPILLON trials, demonstrating clinical benefits. Multiple other ongoing investigations include assessing whether the fourth-generation inhibitors, antibody-drug conjugates, bispecific antibodies, and new combination therapy work through multiple pathways of resistance (Table 5) [53-55].

**Table 5:** Major ongoing and completed clinical trials The table is compiled by the authors [53-55]. EGFR - epidermal growth factor receptor; ALK - anaplastic lymphoma kinase; MET - mesenchymal-epithelial transition factor; TKI - tyrosine kinase inhibitor; PFS - progression-free survival; CNS - central nervous system

Clinical trial	Molecular target	Study design	Major findings	Clinical significance
FLAURA	EGFR	Phase III	Osimertinib superior to first-generation EGFR TKIs	Established first-line osimertinib
AURA	EGFR T790M	Phase I/II	High response after acquired resistance	Approved osimertinib for T790M-positive disease
ALEX	ALK	Phase III	Alectinib superior to crizotinib	Standard first-line ALK therapy
CROWN	ALK	Phase III	Lorlatinib improved PFS and CNS control	Established first-line lorlatinib
MARIPOSA	EGFR + MET	Phase III	Combination therapy improved outcomes	Dual-targeted therapeutic strategy
PAPILLON	EGFR Exon 20	Phase III	Improved efficacy with combination therapy	New option for exon 20 insertion mutations
Various ongoing trials	Fourth-generation EGFR/ALK inhibitors	Phase I–III	Evaluation against resistant mutations	Future resistance-directed therapies

Novel Agents Under Investigation

There are many potentially new-generation, targeted drugs currently in clinical trials. There is some evidence that the fourth-generation EGFR inhibitors are active against the C797S and compound resistance mutations, and new ALK inhibitors (e.g., TPX-0131, NVL-655) are active against complex ALK resistance mutations after lorlatinib failure. Allosteric inhibitors of kinases, proteolysis-targeting chimeras (PROTACs), and mutation-selective inhibitors represent new therapeutic platforms that will continue to increase the availability of therapeutics for resistant disease [54,55].

Combination Therapy Trials

In the clinical research field, using multiple treatment approaches has become a key area for current clinical research. Initial promising data have been elicited using simultaneous targeting of molecules (EGFR and MET) and targeted therapy with antibody-drug conjugates (ADC), or targeted therapy with bispecific antibodies (BsAb). These aim to prevent bypass signaling pathways, slow the onset of resistance, and enhance control of the disease on a longer time scale [55,56].

Precision Medicine Initiatives

Recently, the whole field of precision medicine has gotten a fresh, new, and exciting spin, and multi-omics technologies, sequential liquid biopsy, and AI are now geared towards selecting the right treatment for a patient. With real-time molecular information, the clinician can recognize early on the terrain of resistant mechanisms that reduce the dosage of the unindicated drugs, and thus, along with them the unindicated toxicity, and optimize the therapeutic power of the drug complex employed [56].

Future Directions

In the future, we will witness further developments of therapeutic tools using adaptive treatment algorithms, next-generation highly selective inhibitors, use of AI-based clinical decision support, and combination therapy. Molecular diagnostics in conjunction with computational prediction models will allow for the treatment of individual patients in the future, and may well impact the overall survival of patients with oncogenic-driven NSCLC [56,57].

Challenges and future perspectives

While there have been tremendous advances in targeted treatment for NSCLC, there are also a number of scientific and clinical questions, such as how to achieve long-term control of the disease in patients with EGFR- and ALK- mutated NSCLC. The dynamic evolution of a tumor results in a constant genomic diversification, clonal selection, and adaptation to associated therapeutic pressures. However, it is very challenging to eradicate resistant disease since multiple-resistant subclones of a tumor can co-exist. Therefore, it is hard to imagine that one specifically targeted therapy will ever be developed to silence all of the clones simultaneously, making the use of adaptive approaches and personalized treatment strategies challenging [58].

Changing resistances over time, while changing therapeutic courses, is another major challenge. Several resistance pathways - secondary kinase mutations, activation of bypass signaling pathways, phenotypic transformation, and microenvironment-mediated resistance - can exist concurrently and cause a highly complex resistance profile that needs to be extensively characterized at the molecular level. Whilst next-generation sequencing has now become more widely available, the expensive and molecular profiling process, as well as the lack of availability in many health care facilities, continues to hinder the implementation of precision oncology. Furthermore, disparities in access to new targeted therapies are a major obstacle, particularly in low- and middle-income countries, where access to and coverage of drugs remain a key concern [58,59].

Despite the promise, however, some issues with assay standardization, sensitivity of the analytical procedures, interpretation of low-frequency variants, and how to utilize the procedure in clinical practice are still to be explored. Likewise, the use of artificial intelligence to help predict a treatment's outcome has proven promising in discovering the patterns of resistance and selecting therapy, but such type of computational models need a lot of prospective testing before they can be used routinely in clinics. The multi-omics approach integrates genomic, transcriptomic, proteomic, metabolomic, and epigenomic details, providing a unique chance to gain a greater understanding of resistance biology, but requires complex analysis and a challenging but manageable, multi-dimensional dataset [59].

In precision oncology in the future, the accurate application of personalized adaptive therapy, which is based on molecular monitoring, will be increasingly important, as will be liquid biopsy, AI to provide predictive analytical tools, and real-time analysis of the evolution of the tumor. Continued application of genomic resistance information (GRI), enhancing the development of highly selective fourth-generation inhibitors, as well as antibody-drug conjugates, bispecific antibody therapies and rational combination therapy targeting multiple resistance pathways will substantially boost long term disease control. Finally, further development of a precision oncology roadmap will enable a multidisciplinary approach by clinicians, molecular biologists, bioinformaticians, pharmaceutical scientists and regulatory bodies to translate the initial research findings into clinically successful therapeutic strategies that lead to better outcomes for patients with oncogene-driven NSCLC in terms of survival and quality of life [60].

## Conclusions

Overall, targeted therapy has revolutionized the treatment of EGFR- and ALK-mutated NSCLC and offers patients treatment options that are of high effectiveness and are biomarker driven, improving patients' outcomes. But never a permanent phenomenon, the acquisition of resistance is an inevitable fact and is a product of several secondary mutations, bypassing signaling pathways, phenotypic plasticity, tumoral heterogeneity, and the microenvironment. The sensitivity of resistance detection is becoming increasingly related to novel tumor liquid biopsy, combined tumor-omics analysis, artificial intelligence, and precision medicine, which is increasingly personalized drug therapeutics. Concurrently, innovative strategies to overcome bypass and intrinsic resistance of drugs, including the rational combination of drugs, bispecific antibodies, antibody drug conjugates and fourth-generation kinase inhibitors, are beginning to look like appealing ways of improving clinical benefit. Future research progress in medicine should aim towards adaptive therapy, or one-to-one matching of the right treatment choice with active monitoring of the tumor molecular phenotype. Future research with both translational researchers and clinicians will be crucial to ensure that the new molecular findings are translated into effective clinical interventions and improve survival and life expectancy for those suffering from EGFR- and ALK-mutated NSCLC.
